# Prevalence and heritability of parental‐reported speech and/or language difficulties in a Swedish population‐based twin sample

**DOI:** 10.1002/jcv2.12221

**Published:** 2024-01-31

**Authors:** Rebecka Keijser, Jakob Åsberg Johnels, Marika Habbe, Paul Lichtenstein, Henrik Larsson, Sebastian Lundström, Mark J. Taylor, Kristiina Tammimies

**Affiliations:** ^1^ The Center of Neurodevelopmental Disorders (KIND) Centre for Psychiatry Research Department of Women's and Children's Health Karolinska Institutet and Child and Adolescent Psychiatry Stockholm Health Care Services Stockholm County Council Stockholm Sweden; ^2^ Astrid Lindgren Children's Hospital Karolinska University Hospital Solna Sweden; ^3^ Gillberg Neuropsychiatry Centre Institute of Neuroscience and Physiology University of Gothenburg Gothenburg Sweden; ^4^ Speech and Language Pathology Unit Institute of Neuroscience and Physiology University of Gothenburg Gothenburg Sweden; ^5^ Department of Medical Epidemiology and Biostatistics Karolinska Institutet Stockholm Sweden; ^6^ School of Medical Sciences Örebro University Örebro Sweden

**Keywords:** heritability, language disorder, prevalence, speech disorder, twin study

## Abstract

**Background:**

Research on genetic and environmental influences on speech and/or language difficulties (SaLD) is sparse, with inconsistent heritability estimates. We aimed to estimate the prevalence of parental reported SaLD and the relative contributions of genetic and environmental factors for the phenotype using a Swedish population‐based twin sample. We hypothesized that there would be a stronger genetic than environmental effect on SaLD.

**Methods:**

Data were collected from The Child and Adolescent Twin Study in Sweden. The study sample included 16,774 twin pairs (16,946 males, 16,602 females), of which 5141 were monozygotic, 5861 dizygotic (DZ), and 5772 opposite‐sex DZ pairs. The language items in the Autism–Tics, Attention‐Deficit Hyperactivity Disorder, and other Comorbidities inventory were used to categorize individuals as having parental‐reported SaLD. A classical twin design was used to estimate the relative contribution of genetic and environmental factors to the liability of SaLD.

**Results:**

The prevalence of SaLD was 7.85% (95% confidence interval (CI) [7.57%–8.15%]) and 7.27% (95% CI [6.99%–7.55%]) when excluding individuals with autism and intellectual disability (ID). We also found that SaLD were significantly more prevalent in males than females with a ratio of 2:1. The heritability was estimated to be 75% (95% CI [67%–83%]) for SaLD. Shared environment played a significant role with an estimated contribution of 22% (95% CI [14%–30%]). The heritability estimate was reduced to 70% but with overlapping CI when excluding individuals with autism and ID.

**Conclusions:**

We provide evidence that SaLD is common in the population and under strong genetic influence. Future studies should focus on mapping the genetic architecture of SaLD and related disorders.

## Graphical abstract

Research on genetic and environmental influences on speech and language difficulties is sparse, with inconsistent heritability estimates. We provide robust evidence that speech and language difficulties are common in the population and under strong genetic influences.

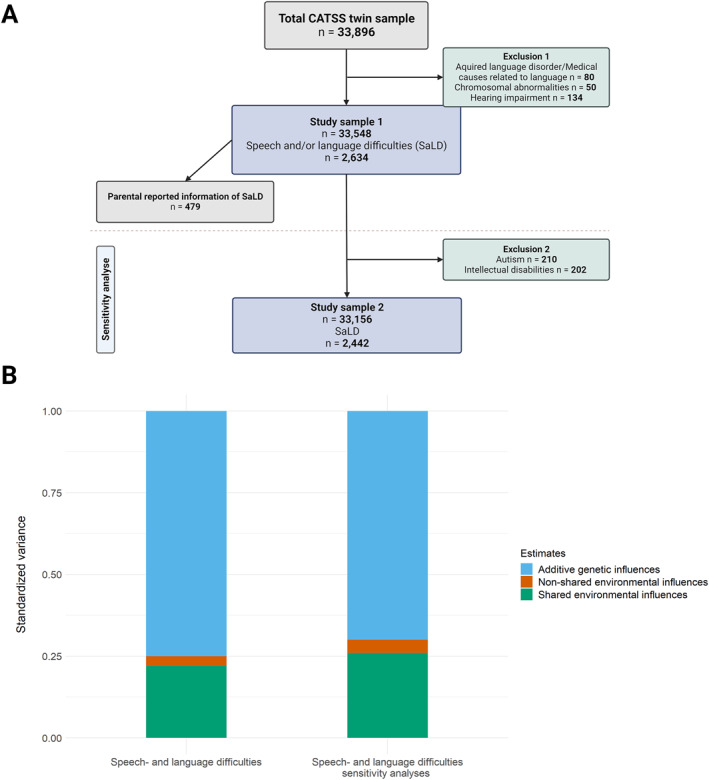

**Key points**

- We reported a prevalence of 7.85% for SaLD in a Swedish population‐based sample, in line with previous studies on speech‐language‐related disorders.
- SaLD were twice as common in males than females.
- We show a strong genetic etiology for the SaLD phenotype in a Swedish population‐based sample. Our results of strong genetic influence on SaLD phenotype motivate future molecular genetic studies.

## INTRODUCTION

The ability to learn and use speech and language has a pivotal role in children's general development, building the base for effective communication, cognitive growth, and social interaction. While speech and language development largely follows a universal pattern (Frank et al., 2017), it is subject to variability in timing among children, which can be largely explained by genetic influences (Hayiou‐Thomas et al., 2012; Verhoef et al., 2021), as well as language‐specific typological features (Bleses et al., 2008).

When a child's ability to speak and understand spoken language develops slower than expected or shows other symptoms differentiating from the typical development, it can be accounted as speech and/or language difficulties (SaLD). Speech and language disorders are diagnosed when the difficulties are beyond a delay and include difficulties in processing, understanding, and/or producing spoken language. The difficulties should also significantly impact daily functioning and often persist throughout life (Botting, 2020; Calder et al., 2022; Clegg et al., 2005; Elbro et al., 2011). For instance, developmental language disorder (DLD) is diagnosed in children when language abilities are significantly below what is expected, affecting their everyday lives and cannot be explained by other biomedical conditions. If the LD is co‐occurring with, for example, intellectual disability (ID) or autism, it is termed language disorder (LD) (Bishop et al., 2017).

Speech and language disorders, while distinct, often demonstrate a high degree of co‐occurrence. For instance, previous studies have reported that 11%–40% of children with speech and sound disorder (SSD) have a co‐occurring DLD (Eadie et al., 2015; Shriberg et al., 1999). In school‐age children with DLD, 66% were reported with SSD (Kalnak et al., 2014).

The prevalence estimates of SaLD and the underlying diagnoses depend on various factors, such as the assessment method (Calder et al., 2022), inclusion criteria (Norbury et al., 2016), or the age of the participants (Nudel et al., 2023). For instance, caregiver‐reported SaLD among approximately 16% of 5‐year‐old children and 8% among 9‐year‐olds (McConkey et al., 2021). On the other hand, a recent population‐based study with over 46,000 adults from Denmark estimated the prevalence of DLD to be 3.7% based on a self‐reported questionnaire (Nudel et al., 2023), compared to the population‐based estimated prevalence of DLD reported in children at 7.5% (Norbury et al., 2016), which was based on direct assessments of language abilities.

Despite the relatively high prevalence of SaLD and the related diagnoses, little is known about the etiology (Mountford et al., 2022). Twin studies have shown a pattern of significant heritability regarding language development (Hayiou‐Thomas et al., 2012; Rice et al., 2014), with greater heritability observed in cases of more severe LD (Spinath et al., 2004).

Classical twin studies using monozygotic (MZ) and dizygotic (DZ) twins have estimated the heritability of language skills at specific ages and longitudinally showing strong genetic influences with a more profound effect among older children in comparison with younger (Hayiou‐Thomas et al., 2012; Kovas et al., 2005; Rice et al., 2018). DeThorne et al. (2006) reported the heritability estimates for SaLD regarding articulation and expressive language difficulties in 248 twin pairs to 0.95 and 0.58, respectively. Bishop and Hayiou‐Thomas (2008) showed heritability estimates of language impairment, using a selected sample of 191 MZ‐ and 193 same‐sex DZ twin pairs, was close to zero when the impairment was based on a language test, and the opposite for twins that were clinically ascertained, with high heritability (0.97), suggesting that the relative importance of genetic and environmental factors differs between variation in language skills and diagnosed speech and language disorders. A recent meta‐analysis for family‐based heritability estimates used 23 studies for communication disorders and provided an overall heritability estimate of 0.69 (Gidziela et al., 2023).

The earlier studies have certain limitations, for example, smaller sample sizes, different methodological approaches, and are often based on samples recruited specially for SaLD instead of population‐based samples (Bishop & Hayiou‐Thomas, 2008; DeThorne et al., 2006; Hayiou‐Thomas et al., 2005; Law et al., 2004). Previous studies estimating the genetic and environmental factors in SaLD, or disorders have been conducted in English‐speaking countries (St Pourcain et al., 2018; Verhoef et al., 2021). To encompass all individuals experiencing difficulties and delays in speech‐ and/or language development and for the purpose of estimating heritability in the general population, we employed the inclusive approach using a parental‐reported SaLD in the present study. We aimed to provide robust prevalence and heritability estimates for SaLD. We hypothesized that using a classical twin design and a large Swedish population‐based sample of over 16,500 twin pairs would clarify the earlier inconsistent findings in heritability estimates for SaLD.

## MATERIALS AND METHODS

### Overview and sample

The Child and Adolescent Twin Study in Sweden (CATSS) is an ongoing population‐based longitudinal twin study of somatic‐ and mental health, personality development, psychosocial adaptation, and biological data and uses a range of data collection methods, for example, interviews, questionnaires, and DNA–sampling with a response rate of 70%. Parents of twins born in Sweden since 1992 are contacted around the twins' 9^th^ or 12^th^ birthdays. The total sample used for this study consisted of 16,633 twin pairs with known zygosity (Table 1). Zygosity was assessed through 48 single nucleotide polymorphisms (SNPs) (Hannelius et al., 2007), SNP microarray (Zagai et al., 2019), or by an algorithm assessing twin similarity (Anckarsater et al., 2011). The CATSS has been thoroughly described earlier (Anckarsater et al., 2011; Taylor et al., 2020, 2023).

**TABLE 1:** Sample characteristics.

Variable	Sample distribution, N (%)			Distribution of twins, N (%)					
	Total (%)	Males (%)	Females (%)	MZM (%)	MZF (%)	DZM (%)	DZF (%)	DZOSM (%)	DZOSF (%)
Twins	33,548	16,946	16,602	4930	5352	6244	5478	5772	5772
SaLD	2634 (7.85)	1798 (10.61)	836 (5.03)	696 (14.11)	315 (5.89)	582 (9.32)	247 (4.50)	520 (9.00)	274 (4.74)
Autism	210 (0.63)	149 (0.88)	61 (0.37)	35 (0.71)	15 (0.28)	63 (1.00)	20 (0.37)	51 (0.88)	26 (0.45)
Intellectual disability	202 (0.60)	132 (0.78)	70 (0.42)	33 (0.67)	19 (0.35)	54 (0.86)	27 (0.49)	45 (0.78)	24 (0.4)

*Note*: SaLD = speech and/or language difficulties; All characteristics are calculated post exclusion of Hearing impairment, acquired language disorder and Chromosomal abnormalities; MZ = monozygotic, DZ = dizygotic, DZOS = dizygotic opposite sex; MZM = MZ male; MZF = MZ female; DZM = DZ male; DZF = DZ female; DZOSM = DZOS male; DZOSF = DZOS female.

### Ethical consideration

Participants in CATSS are protected by the informed consent process (Anckarsater et al., 2011). Child and Adolescent Twin Study in Sweden has ethical approval from the Ethical Review Board of Region Stockholm.

### Measures

#### Speech and/or language difficulties phenotype

**FIGURE 1:**
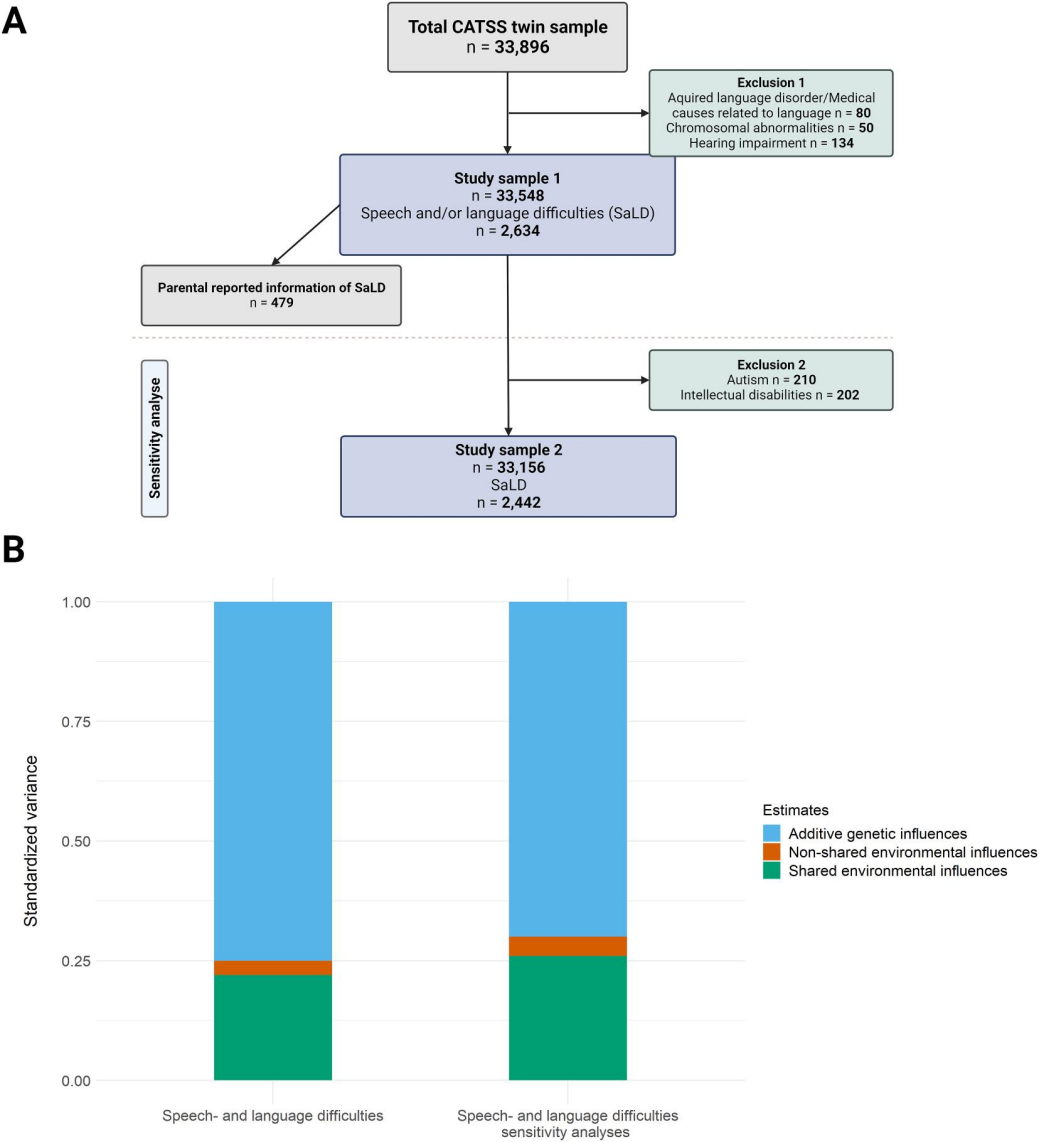
(A) Flowchart of the inclusion and exclusion criteria and the sample presented as the number of participants used for models 1 and 2. Created in BioRender.com. (B) The proportions of variance explained from the best‐fitting ACE models 1 and 2 for speech and language difficulties (SaLD) and SaLD with the exclusion of autism and intellectual disabilities. A = additive genetic; C = shared (or “common”) environment; E = non‐shared environment and the measurement error.

The overview of the inclusion and exclusion criteria for SaLD and the models are presented in Figure 1A. We used the parental reports on the screening instrument Autism‐Tics, AD/HD, and other Comorbidities inventory (A‐TAC) to define individuals with SaLD (Marland et al., 2017). The A‐TAC comprises 96 questions on neurodevelopmental symptoms, including several language items (Hansson et al., 2005; Marland et al., 2017). The A‐TAC has good reliability and validity for most conditions it is screening for (Hansson et al., 2005; Marland et al., 2017).

The questions are administered to parents when their children are either 9‐years‐old (*n* = 26,274) or 12‐years‐old (*n* = 7622). We specifically used the following question to define SaLD in this study: *Was he or she late in learning to speak or talk? The question was followed with an example: If one does not begin speaking by the age of 4‐5, one is considered late, significant problems in speaking count.* The response scale is the following: ‘No’ (0); ‘Yes, to a certain degree’ (1); ‘Yes’ (2).

Individual twins whose parents answered ‘yes’ or ‘yes, to a certain degree’ were categorized as having SaLD (Model 1). Furthermore, a follow‐up questions administered to the parents who answers or ‘Yes, to a certain degree’ or ‘Yes’: Was *his/her language development delayed, or does s/he not speak at all due to an impairment? If yes, please specify the impairment.* The response options were ‘No’ (0), ‘Autism/autism disorder/Asperger’ (1), ‘Yes (2)’. Parents who endorsed the question by answering ‘yes’ were directed to an open‐ended question ‘*If yes, please state which impairment’*. Initially, we employed the question as a resource to exclude individuals with acquired language disorders (e.g. cerebral palsy, brain injury, epilepsy) or hearing disabilities to avoid potential differential misclassification. Subsequently, we grouped the reported causes into speech/language related diagnosis categories, or non‐specified causes. The non‐specified category included also insufficient responses such “kindergarten teacher” or “ear wax”, and the non‐relevant responses were, for example, “impaired vison” or “heart problems”.

Two clinical validation studies have been conducted within CATSS, described elsewhere (Larson et al., 2013; Åsberg Johnels et al., 2021). Briefly, twin pairs where one or both twins were screened positive for neurodevelopmental disorders (NDDs) and control pairs without any indication of NDDs were invited to participate in the studies. The study protocol included, among other things, the full Wechsler Intelligence Scales for Children, 4^th^ edition (WISC‐IV) (Wechsler & Kodama, 1949), which provides norm‐referenced scores for specific cognitive domains (all expressed in IQ scores around a normative *M* = 100, SD = 15), including a verbal comprehension index.

#### Other measurements

Autism diagnosis, intellectual disabilities (ID) and chromosomal abnormalities were retrieved from the Swedish National Patient Register (NPR), maintained by the Swedish National Board of Health and Welfare (National Patient Register, 2022) (Supplementary Table 1). All conditions were coded as absent = 0; present = 1. Additionally, children of parents who responded ‘Autism/autism disorder/Asperger’ to the follow‐up question of SaLD were clustered together with NPR diagnosis of autism and used as a single variable.

### Statistical analyses

#### Sex differences in prevalence estimates

We used the χ^2^ test to analyze differences in the prevalence of SaLD between males and females in the sample.

#### Twin design

The classical twin design aims to estimate the relative contribution of genetic and environmental factors to liability to a disorder. We started by calculating tetrachoric twin correlations to assess twin resemblance on SaLD phenotype. Variance in liability underlying SaLD are assumed to be driven by additive genetic (A), that is, genes whose allelic effects combine additively, shared (or ‘common’) environment (C), which includes all environmental factors increasing similarity between the twins, and non‐shared environment I, which includes environmental exposures not shared by co‐twins and measurement error (Neale & Cardon, 1992). Tetrachoric twin correlations are calculated for dichotomized phenotypes, for example, absent or present, indicating similarity between twin pairs and providing information regarding genetic effects (Olsson, 1979). When the correlation of MZ twins is higher than that of the DZ twins, it is assumed that genetic variation influences individual differences in liability to SaLD phenotype. A shared environment is indicated when the MZ and DZ correlations are more similar. Non‐shared environmental influences are indicated by how the MZ correlation differs from 1 (Prescott & Kendler, 1995). The similarity in MZ twins is due to identical genes, that is, A and shared environment, C, while DZ twins share approximately 50% of their A and 100% of C (Neale & Cardon, 1992).

#### Twin models

We first fitted a fully saturated model, a reference model of the observed data. We used this model to test assumptions that thresholds (i.e., prevalence) are equal within twin pairs and across zygosity and to estimate tetrachoric correlations. After that, we fitted univariate liability threshold models. Liability threshold models assume that a normal distribution of genetic and environmental risk factors underlies categorical variables (i.e., liability). The model estimates the proportion of variation in liability that is genetic (i.e., heritability) and environmental in the population. Univariate liability threshold ACE models were used to yield estimates of the relative contributions of A, C, and E to variance in liability to SaLD (Model 1). To test whether potential heritability estimates were influenced by the inclusion of individuals diagnosed with autism or ID, individuals with these conditions were excluded, and we, again, fitted a univariate liability threshold ACE model for SaLD (Model 2).

Model fit was assessed by calculating the difference between the ACE models' negative log‐likelihood (‐2LL) and saturated models. The difference in ‐2LL is an asymptotically distributed *χ* ^2^ with degrees of freedom (*df*) equal to the difference in the number of parameters in both models.

All analyses were conducted in the statistical program R version 4.2.0 (R Core Team, 2022). The package used for twin modeling was the OpenMX 2.0 package (Neale et al., 2016).

## RESULTS

Descriptive statistics of the study sample are provided in Table 1 and Figure 1A. The study sample included in the analyses, after exclusion criteria, consisted of 16,774 twin pairs (9 years old = 12,985 twin pairs, 12‐year‐old = 3789 twin pairs), including 5141 MZ pairs 5861 DZ pairs and 5772 opposite‐sex DZ pairs. The sample also included individuals diagnosed with autism or ID, later excluded for sensitivity analyses.

### Prevalence of speech and/or language difficulties

The prevalence estimates for autism, ID and SaLD are presented in Table 1. Based on the parental report, 7.85% (95% CI [7.57% – 8.15%], *n* = 2634) of the twins had SaLD. When excluding individuals with autism and/or ID, the prevalence was 7.27% (95% CI [6.99% – 7.55], *n* = 2424). The prevalence in males was 10.61% (*n* = 1798) and 5.03% (*n* = 836) for females, indicating a male‐to‐female ratio of 2:1 (OR = 2.23, 95% CI [2.05 – 2.43]), with a significant difference between the prevalence (*χ* ^*2*^(1, *n* = 2634) = 360.21, *p* < 0.001).

Of the 2424 children reported with SaLD, 512 parents indicated the SaLD were due to an impairment related to a known cause or diagnosis, and of these 479 responded to the open‐ended follow‐up question (Table 2). Among these 479 children, DLD was reported in 19.8%, speech sound disorder in 13.7%, and 3.5% had DLD and/or SSD (Table 2). Additionally, 31 parents (6.5%) had indicated autism as the reason behind SaLD.

**TABLE 2:** Categories of parent's comments on their child's Speech and/or language difficulties (SaLD) in 512 twins.

	Total (%)	Male (%)	Female (%)
Parental reported information on SaLD	479 (93.5)^a^	331 (69.1)	148 (30.9)
Developmental language disorder	95 (19.8)	69 (20.8)	26 (17.6)
Language disorder (due to ID or Autism)^b^	87 (18.1)	54 (16.3)	33 (22.3)
Hearing impairment^c^	83 (17.3)	58 (17.5)	25 (16.8)
Acquired language disorder/Medical causes related to language^c^	80 (16.7)	49 (14.8)	31 (20.9)
Speech sound disorder	66 (13.7)	54 (16.3)	12 (8.1)
Not specified	51 (10.6)	35 (10.5)	16 (10.8)
Developmental language disorder/Speech sound disorder	17 (3.5)	12 (3.6)	5 (3.3)

^a^ Parental reported information on SaLD from 512 individuals that indicated impairment/known diagnosis for the difficulties. For 33 individuals no open‐ended answers were recorded.

^b^ Removed in sensitivity analyses.

^c^ Hearing impairment and Acquired LD were both removed from the main analysis to avoid potential differential misclassification of SaLD.

As the defined SaLD was based on parental report, we investigated differences in quantitative measures of SaLD in a clinical subsample within CATSS by comparing cognitive test scores of children whose parents answered: ‘yes’ or ‘yes, to a certain degree’ on the first SaLD item (*n* = 66) and those whose parents answered ‘no’ (*n* = 377). The children with SaLD based on the parental report had a mean verbal comprehension WISC‐IV score of 81.5 (SD = 19.6) while children without SaLD had a mean score of 98.3 (SD = 21.4), *t*(441) = 5.95, *p* < 0.001. The corresponding numbers when excluding individuals with autism and/or ID were 89.5 (SD = 18.0, *n* = 36) and 101.0 (SD = 19.9, *n* = 301) for the SaLD group and the controls, respectively (*t*[335] = 3.31, *p* < 0.001). By contrast, the group with reported SaLD had a mean (nonverbal) perceptual reasoning score of 98.3 (SD = 11.9, *n* = 36), that is, close to the normative mean of 100. Thus, the language item used for the initial group assignment in the current study seems to identify children with weak verbal development and have some discriminant validity related to broader aspects of cognitive development.

### Twin analyses

We first calculated the twin correlations for SaLD. Greater tetrachoric correlations were found for MZ twins, with a correlation of 0.97 (95% CI [0.96 – 0.98]) compared with a DZ correlation of 0.58 (95% CI [0.54 – 0.62]) for SaLD. The same pattern was seen in the sensitivity analyses with MZ correlation of 0.97, 95% CI [0.97 – 0.98], compared with DZ estimates of 0.60, 95% CI [0.56 – 0.64]. These findings suggest that genetic factors influenced individual differences in the liability to SaLD.

Next, we fitted the twin model to the data for SaLD (Model 1, Table 3). The findings from Model 1 pointed toward a full ACE model. The proportions of variance explained by A, C, and E in liability to SaLD are presented in Figure 1B. For SaLD, the heritability was estimated to be A = 0.75 (95% CI [0.67 – 0.83]), and the remaining variation was accounted for mainly by shared (C = 0.22, 95% CI [0.14 – 0.30]) and a small proportion by non‐shared environment (E = 0.03, 95% CI [0.02 – 0.04]).

**TABLE 3:** Twin model fit statistics.

	Comparison	‐2LL	*df*	Parameters	∆χ^2^	∆*df*	*P*
Model 1; SaLD							
Saturated	‐	15,808.87	33,456	6	‐	‐	‐
ACE	Saturated	15,865.99	33,550	4	57.11	4	<0.001
AE	ACE	15,891.42	33,551	3	25.44	1	<0.001
CE	ACE	16,286.73	33,551	3	395.30	1	<0.001
E	ACE	18,459.85	33,552	2	2173.12	2	<0.001
Model 2; SaLD – sensitivity analyses							
Saturated	‐	14,472.51	32,876	6	‐	‐	‐
ACE	Saturated	14,542.02	32,880	4	69.51	4	<0.001
AE	ACE	14,574.23	32,881	3	32.21	1	<0.001
CE	ACE	14,907.67	32,881	3	333.44	1	<0.001
E	ACE	17,025.58	32,882	2	2117.91	2	<0.001

*Note*: SaLD = speech and/or language difficulties; A = additive genetic; C = shared (or “common”) environment; E = non‐shared environment and the measurement error; ‐2LL = fit statistics −2*log‐likelihood of the data; *df* = degrees of freedom; ∆χ^2^ = change in ‐2LL between two models, distributed χ^2^; ∆*df* = change in degrees of freedom between two models.

In the second step, we analyzed the possible effects of autism and ID diagnoses on the heritability estimates of SaLD phenotype (Figure 1B). The finding of the sensitivity analyses followed the same pattern where Model 2 pointed toward a full ACE model. The estimates were slightly decreased, with SaLD heritability estimated to be A = 0.70 (95% CI [0.62 – 0.79]), and the remaining variation was explained by shared (C = 0.26, 95% CI [0.17 – 0.34]) and non‐shared environment (E = 0.03, 95% CI [0.02 – 0.04]). The findings from the sensitivity analysis show that the inclusion of individuals with autism or ID in the study did not strongly influence our estimates.

## DISCUSSION

We show a prevalence of parental‐reported SaLD of 7.85% in this Swedish population‐based twin sample. The prevalence is lower than in two previous studies in English‐speaking population‐based screening studies among 5 and 9 ‐year‐old (McConkey et al., 2021; Norbury et al., 2016) but higher than a recent population‐screening among adults in Denmark (Nudel et al., 2023). Most speech‐ and language‐related diagnoses are not included in the NPR that have provided excellent coverage to study other NDDs (Martini et al., 2022; Taylor et al., 2020). Therefore, our selection of the overarching term SaLD was motivated to maximize sensitivity, ensuring the inclusion of all potential speech and/or language‐related difficulties within a general population, including related causes as verified in Table 2. We also report the validity of the parental report data from a clinical sample, suggesting construct validity. Most likely, if clinically assessed, many children here classified as having SaLD would also have a speech and/or LD diagnosis.

The prevalence of NDD is often male‐biased (Loomes et al., 2017; May et al., 2019); we show the same pattern of estimates, with a male‐to‐female ratio of 2:1 for which showed a significant pattern, in line with a previous population‐based sample of children with SaLD (McConkey et al., 2021). Interestingly, earlier studies have shown that clinical samples differ from population‐based ones regarding sex differences, with the clinically ascertained samples having a significantly higher prevalence of language disorders among males (Hobson & Bird, 2019). For other NDD, the higher prevalence among males in clinical samples has been suggested to be due to females not receiving a diagnosis, despite meeting the diagnostic criteria (Loomes et al., 2017). Further studies with even larger sample sizes should focus on differences in SaLD based on sex and investigate whether sex‐specific patterns exist in its etiology.

Our findings in the study clearly show that SaLD are under strong genetic influences. The heritability estimates were slightly reduced in our sensitivity analyses when excluding participants with autism and ID diagnosis, even though, still high. Although some inconsistencies can come from criteria and definitions of language phenotypes related to development or impairment (Hobson & Bird, 2019), earlier studies have been small in sample size. Here, we provide robust estimates from a large well‐defined twin sample and add a screening measure for SaLD among the highly heritable conditions under the umbrella of NDD.

Our results of strong genetic influence on SaLD motivate future molecular genetic studies in the area that so far has been sparse (Deriziotis & Fisher, 2017; Mountford et al., 2022). There are already known monogenic and genomic disorders that are found to cause speech and language disorders, but these account for only a small fraction of the cases (Deriziotis & Fisher, 2017; Kalnak et al., 2018; Mountford et al., 2022). All whole exome and genome sequencing studies for these phenotypes have small sample sizes (Andres et al., 2021, 2023; Eising et al., 2019; Hildebrand et al., 2020; Kornilov et al., 2016; Mountford et al., 2022); thus, larger studies are needed to find new genes and genetic variations contributing to speech‐ and/or language developmental difficulties and subsequent disorders.

As rare genetic disorders only explain a small portion of the genetic architecture of these phenotypes, other genetic factors, that is, common SNPs, likely play a key role in aetiology. Genome‐wide association studies to identify the associated SNPs for speech‐ and/or language‐disorders, are still few, with limited evidence for genome‐wide significant variants (Eising et al., 2022; Nudel et al., 2014, 2023). The recent meta‐analysis Genome‐wide association studies studies with much larger sample sizes for reading and language traits have shown genome‐wide significant hits (Doust et al., 2022; Eising et al., 2022). Future molecular genetic studies focusing on the SaLD in large samples are needed to understand the full landscape of genetic variation associated with the phenotype.

Although genetic influences are strong for SaLD, environmental factors play an important role, especially shared environment. The recent meta‐analysis of heritability estimates of communication disorders also showed significant shared environmental influence (*C* = 0.36) (Gidziela et al., 2023). Previous studies have shown that various environmental factors may influence the susceptibility of SaLD, such as low parental education and inadequate stimulation (Sunderajan & Kanhere, 2019). Further environmental risk factors have been suggested; older maternal age at the child's birth, introverted paternal personality, low monthly family income, and lack of parent–child communication (Fan et al., 2021). There is a lack of research focusing on environmental factors associated with SaLD, especially compared to studies of other NDDs (Carlsson et al., 2021). It is also largely unknown whether the reported associations between environmental factors and the difficulties reflect a causal link between these exposures and SaLD, or if unmeasured familial confounding plays a role (Carlsson et al., 2021). Given the high heritability of SaLD reported in this study, and the fact that many environmental exposures are to some degree heritable, there is a need for genetically informative studies to test whether environmental factors are a cause of SaLD or whether their association reflects shared genetics.

Our study has several strengths, including a population‐based, genetically informative large sample. The classical twin design is a well‐known approach with great statistical power when the aim is to evaluate heritability estimates and differentiate between genetic and shared/nonshared environmental influences (Hagenbeek et al., 2023). Other genetic statistical approaches are more suitable for estimates of genetic correlations and associations.

We also acknowledge several limitations in our study. Firstly, the terminology SaLD does not signify a clinical diagnosis of DLD or other speech and/or language‐related diagnoses. Rather, it serves as an inclusive measure encompassing a diverse spectrum of SaLD, some of which may have a known underlying diagnosis, while others may not. As this is a screening approach, it cannot be used to estimate heritability for specific diagnoses, nor does it detail etiological differences in those with “only” speech or “only” language‐related difficulties.

Moreover, the use of parental reports may affect the credibility of the prevalence estimates due to recall bias (Infante‐Rivard & Jacques, 2000). However, parental reports are often quite accurate and widely used in research in general (Conway et al., 1980), including in SaLD research (Feeney et al., 2012, 2016, 2017; Hobson et al., 2022; Markham & Dean, 2006; McConkey et al., 2021).

## CONCLUSION

The SaLD phenotype in the current study is common in the population and highly heritable, providing persistent information on the architecture of SaLD etiology. However, the genetic and environmental contribution to SaLD is not yet understood, thus calling for more studies to understand the causes.

## AUTHOR CONTRIBUTIONS

**Rebecka Keijser**: Conceptualization; Data curation; Formal analysis; Investigation; Methodology; Project administration; Software; Validation; Visualization; Writing – original draft; Writing – review & editing. **Jakob Åsberg Johnels**: Data curation; Investigation; Methodology; Writing – review & editing. **Marika Habbe**: Investigation; Methodology; Writing – review & editing. **Paul Lichtenstein**: Data curation; Methodology; Writing – review & editing. **Henrik Larsson**: Data curation; Methodology; Writing – review & editing. **Sebastian Lundström**: Data curation; Methodology; Writing – review & editing. **Mark J. Taylor**: Conceptualization; Data curation; Formal analysis; Funding acquisition; Investigation; Methodology; Project administration; Resources; Software; Supervision; Writing – review & editing. **Kristiina Tammimies**: Conceptualization; Data curation; Funding acquisition; Investigation; Methodology; Project administration; Resources; Software; Supervision; Writing – review & editing.

## CONFLICT OF INTEREST STATEMENT

Henrik Larsson reports receiving grants from Shire Pharmaceuticals; personal fees from and serving as a speaker for Medice, Shire/Takeda Pharmaceuticals, and Evolan Pharma AB; and sponsorship for a conference on attention‐deficit/hyperactivity disorder from Shire/Takeda Pharmaceuticals and Evolan Pharma AB, all outside the submitted work. Henrik Larsson is editor‐in‐chief of JCPP *Advances*. The remaining authors have declared that they have no competing or potential conflicts of interest.

## ETHICAL CONSIDERATIONS

Participants in CATSS are protected by the informed consent process (Anckarsater et al., 2011). Child and Adolescent Twin Study in Sweden has ethical approval from the Ethical Review Board of Region Stockholm.

## Supporting information

Supplementary Material

## Data Availability

The raw data is available through the Swedish Twin Registry (https://ki.se/en/research/swedish‐twin‐registry‐for‐researchers). The code used to generate the correlations and models and generate figures are publicly available on the GitHub page for the study: https://github.com/Tammimies‐Lab/LanguageDelay_heritability_Keijser.
