# Supplementary material for: Prevalence and heritability of parental‐reported speech and/or language difficulties in a Swedish population‐based twin sample
Source: JCPP Adv. 2024 Jan 31;4(3):e12221. doi: 10.1002/jcv2.12221 (PMC11472810; doi:10.1002/jcv2.12221)
Supplement: Supplementary file 1 — Supplementary Material [file JCV2-4-e12221-s001.docx]

*Keijser et al. 2024 SaLD*

| **Supplementary Table 1.** International Statistical Classification of Diseases and Related Health Problems 10^th^ Revision codes over study variables | | |
| --- | --- | --- |
| **Classification** | **Diagnosis** | **Code** |
| **Pervasive developmental disorders** | Childhood autism | F84.0 |
|  | Atypical autism | F84.1 |
|  | Asperger syndrome | F84.5 |
|  | Other pervasive developmental disorders | F84.8 |
|  | Pervasive developmental disorder, unspecified | F84.9 |
| **Mental retardation** |  |  |
|  | Mild mental retardation | F70 |
|  | Moderate mental retardation | F71 |
|  | Severe mental retardation | F72 |
|  | Profound mental retardation | F73 |
|  | Other mental retardation | F78 |
|  | Unspecified mental retardation | F79 |
| **Chromosomal abnormalities, not elsewhere classified** |  |  |
|  | Down syndrome | Q90 |
|  | Edwards syndrome and Patau syndrome | Q91 |
|  | Other trisomies and partial trisomies of the autosomes, not elsewhere classified | Q92 |
|  | Monosomies and deletions from the autosomes, not elsewhere classified | Q93 |
|  | Balanced rearrangements and structural markers, not elsewhere classified | Q95 |
|  | Turner syndrome | Q96 |
|  | Other sex chromosome abnormalities, female phenotype, not elsewhere classified | Q97 |
|  | Other sex chromosome abnormalities, male phenotype, not elsewhere classified | Q98 |
|  | Other chromosome abnormalities, not elsewhere classified | Q99 |
| Note. Retrieved from the National Patient Register (NPR) | | |
